# Supplementary material for: Mitochondrial activity promotes neutrophil degranulation and endothelial dysfunction in systemic infections
Source: EMBO Mol Med. 2026 May 27;18(7):2691–722. doi: 10.1038/s44321-026-00453-1 (PMC13365472; doi:10.1038/s44321-026-00453-1)
Supplement: Supplementary file 1 — Table EV1 [file 44321_2026_453_MOESM1_ESM.docx]

**Table EV1**. Exact p-values for each figure.

| **Fig. 1A** | |
| --- | --- |
| GEnC:unstim vs. GEnC:A23187 | 0.1621 |
| GEnC+neutrophils:unstim vs. GEnC+neutrophils:A23187 | <0.0001 |
| Neutrophils:unstim vs. Neutrophils:A23187 | >0.9999 |
| GEnC:unstim vs. GEnC+neutrophils:unstim | 0.3113 |
| GEnC:unstim vs. GEnC+neutrophils:A23187 | <0.0001 |
| **Fig. 1B** | |
| endothelium vs. NCM unstim | 0.0106 |
| endothelium vs. NCM TNFα | <0.0001 |
| endothelium vs. NCM A23187 | <0.0001 |
| **Fig. 1C** | |
| unstim vs. NE | 0.0005 |
| unstim vs. PR3 | 0.9994 |
| unstim vs. NE+PR3 | <0.0001 |
| **Fig. 3E** | |
| HC vs. uncompl. malaria | 0.6104 |
| HC vs. severe malaria | 0.0037 |
| **Fig. 3F** | |
| HC vs. sepsis | 0.0007 |
| **Fig. 3G** | |
| Ly6G^+^CD101^-^ vs. Ly6G^+^CD101^+^ | 0.0352 |
| **Fig. 3H** | |
| CD101^+^ vs. CD101^-^ (glycolysis) | 0.0266 |
| CD101^+^ vs. CD101^-^ (mitochondria) | 0.0413 |
| **Fig. 3J** | |
| CTR vs. GCSF-D (vehicle) | 0.7562 |
| CTR vs. GCSF-D (fMLP) | 0.0191 |
| CTR vs. GCSF-D (H) | 0.0389 |
| CTR vs. GCSF-D (A23187) | 0.2903 |
| **Fig. 4D** | |
| HC vs. malaria | 0.9825 |
| HC vs. sepsis | 0.3142 |
| HC vs. GCSF-D | 0.1687 |
| **Fig. 4E** | |
| HC vs. malaria | <0.0001 |
| HC vs. sepsis | 0.9945 |
| HC vs. GCSF-D | 0.0335 |
| **Fig. 4F** | |
| HC vs. malaria | 0.0002 |
| HC vs. sepsis | 0.9889 |
| HC vs. GCSF-D | >0.9999 |
| **Fig. 5A** | |
| uninfected vs. Day 8 | 0.0149 |
| uninfected vs. Day 9 | 0.0006 |
| **Fig. 5B** | |
| HC vs. sepsis | 0.0286 |
| **Fig. 5C** | |
| CTR vs. GCSF-D | 0.0002 |
| **Fig. 5D** | |
| unstim:vehicle vs. A23187:vehicle | <0.0001 |
| unstim:SkQ1 vs. A23187:SkQ1 | 0.0819 |
| unstim:NecroX-5 vs. A23187:NecroX-5 | 0.1284 |
| A23187:vehicle vs. A23187:SkQ1 | 0.0009 |
| A23187:vehicle vs. A23187:NecroX-5 | 0.0946 |
| A23187:SkQ1 vs. A23187:NecroX-5 | 0.9736 |
| **Fig. 5E** | |
| unstim:vehicle vs. unstim:SkQ1 | 0.3862 |
| unstim:vehicle vs. H/T:vehicle | 0.0084 |
| H/T:vehicle vs. H/T:SkQ1 | 0.0230 |
| unstim:SkQ1 vs. H/T:SkQ1 | 0.2082 |
| **Fig. 5F** | |
| unstim:vehicle vs. unstim:SkQ1 | 0.8715 |
| A23187:vehicle vs. A23187:SkQ1 | 0.3716 |
| unstim:vehicle vs. A23187:vehicle | 0.0272 |
| unstim:vehicle vs. A23187:SkQ1 | 0.5604 |
| **Fig. 5G** | |
| unstim:vehicle vs. unstim:SkQ1 | >0.9999 |
| unstim:vehicle vs. A23187:SkQ1 | 0.0001 |
| A23187:vehicle vs. A23187:SkQ1 | 0.0486 |
| unstim:vehicle vs. A23187:SkQ1 | 0.0909 |
| **Fig. 5J** | |
| PMA:vehicle vs. PMA:SkQ1 | 0.0505 |
| A23187:vehicle vs. A23187:SkQ1 | <0.0001 |
| **Fig. 6C** | |
| vehicle vs. SkQ1 (HC) | 0.0328 |
| vehicle vs. SkQ1 (sepsis) | 0.1561 |
|  | |
| **Fig. EV1A** | |
| GEnC, :unstim vs. GEnC, neutrophils:unstim | 0.0201 |
| GEnC, :unstim vs. GEnC, neutrophils:TNFα | 0.0003 |
| **Fig. EV1C** | |
| baseline vs. 5.00 μg/mL | <0.0001 |
| **Fig. EV1D** | |
| baseline vs. 20.00 μg/mL | 0.0014 |
| baseline vs. NS1 (5.00 μg/mL) | 0.0477 |
| **Fig. EV1E** | |
| unstim NCM+PMNs vs. stim NCM+PMNs | 0.0001 |
| stim NCM+PMNs vs. stim NCM+NEi+CGi+PMNs | 0.0100 |
| unstim NCM+PMNs vs. stim NCM+CGi+PMNs | 0.0001 |
| **Fig. EV2H** | |
| HC vs. sepsis | 0.0021 |
| **Fig. EV3C** | |
| uninfected vs. *P. chabaudi* | 0.0337 |
| **Fig. EV3D** | |
| uninfected vs. *P. chabaudi* | 0.0253 |
| **Fig. EV3G** | |
| uninfected vs. Day 9 | <0.0001 |
| uninfected vs. Day 11 | 0.0006 |
| **Fig. EV3H** | |
| uninfected vs. Day 9 | <0.0001 |
| uninfected vs. Day 11 | <0.0001 |
| uninfected vs. Day 13 | <0.0001 |
| **Fig. EV3I** | |
| uninfected vs. *P. chabaudi* CB (blood) | <0.0001 |
| uninfected vs. *P. chabaudi* CB (spleen) | <0.0001 |
| **Fig. EV3K** | |
| CTR vs. GCSF-D | 0.0245 |
| **Fig. EV3M** | |
| CTR vs. GCSF-D | 0.0143 |
| **Fig. EV5D** | |
| vehicle vs. SkQ1 [500 nM] | 0.0013 |
| **Fig. EV5E** | |
| vehicle:A23187 vs. SkQ1:A23187 | <0.0001 |
| **Fig. EV5F** | |
| vehicle:H vs. SkQ1:H | 0.0354 |
| Fig. EV5G | |
| vehicle:unstim vs. vehicle:PMA | <0.0001 |
| **Fig. EV5J** | |
| vehicle vs. SkQ1 (LPS) | 0.0008 |
| vehicle vs. SkQ1 (resiquimod) | <0.0001 |
| **Fig. EV5K** | |
| vehicle vs. SkQ1 (LPS) | 0.0011 |
| vehicle vs. SkQ1 (resiquimod) | <0.0001 |
