# Supplementary figures and images for: Mitochondrial activity promotes neutrophil degranulation and endothelial dysfunction in systemic infections
Source: EMBO Mol Med. 2026 May 27;18(7):2691–722. doi: 10.1038/s44321-026-00453-1 (PMC13365472; doi:10.1038/s44321-026-00453-1)

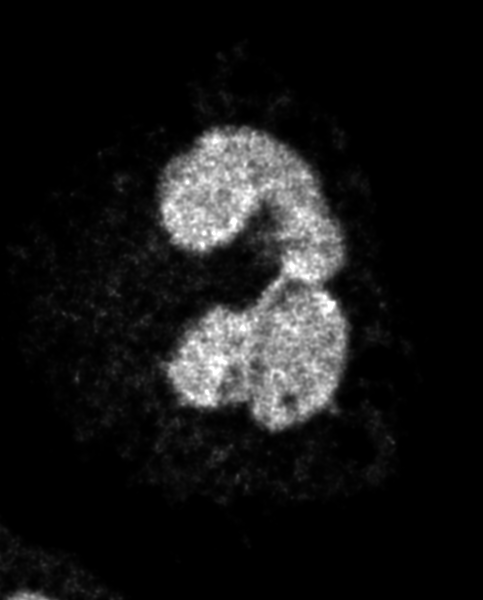

Supplement: Supplementary file 5 — Source data Fig. 4 [file 44321_2026_453_MOESM5_ESM.zip › Figure 4/4A/HC for malaria mitotracker.tif]

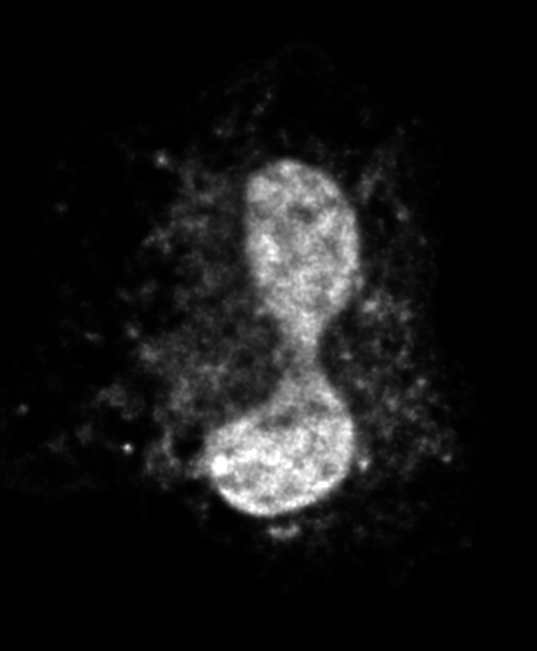

Supplement: Supplementary file 5 — Source data Fig. 4 [file 44321_2026_453_MOESM5_ESM.zip › Figure 4/4A/malaria mitotracker.tif]

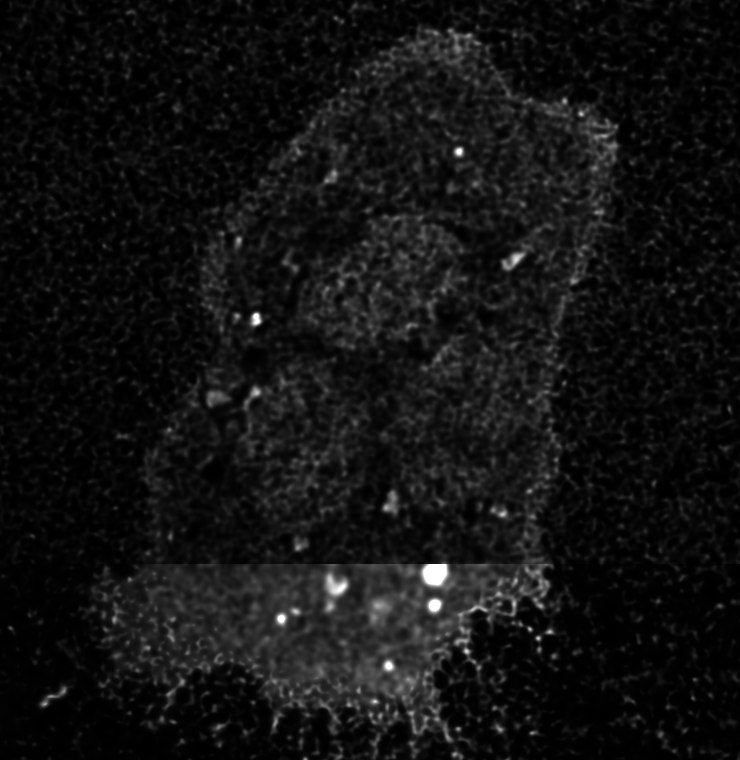

Supplement: Supplementary file 5 — Source data Fig. 4 [file 44321_2026_453_MOESM5_ESM.zip › Figure 4/4B/HC for sepsis mitotracker.tif]

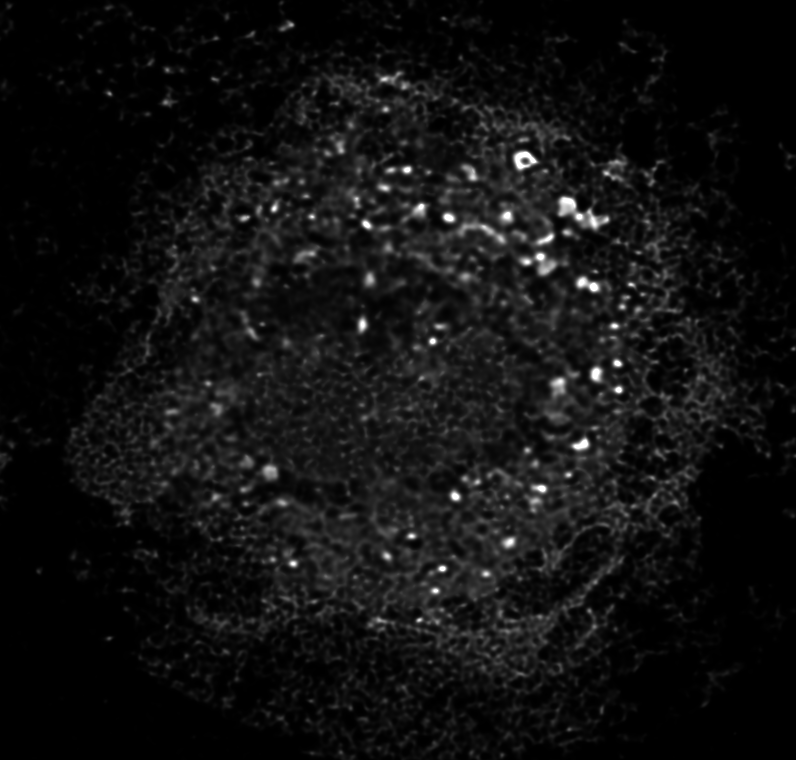

Supplement: Supplementary file 5 — Source data Fig. 4 [file 44321_2026_453_MOESM5_ESM.zip › Figure 4/4B/sepsis mitotracker.tif]

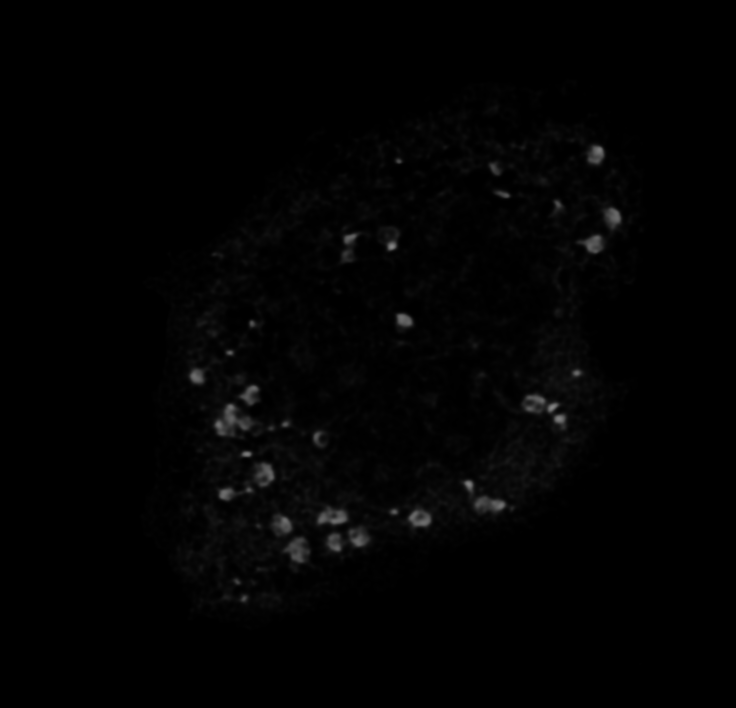

Supplement: Supplementary file 5 — Source data Fig. 4 [file 44321_2026_453_MOESM5_ESM.zip › Figure 4/4C/CTR mitotracker.tif]

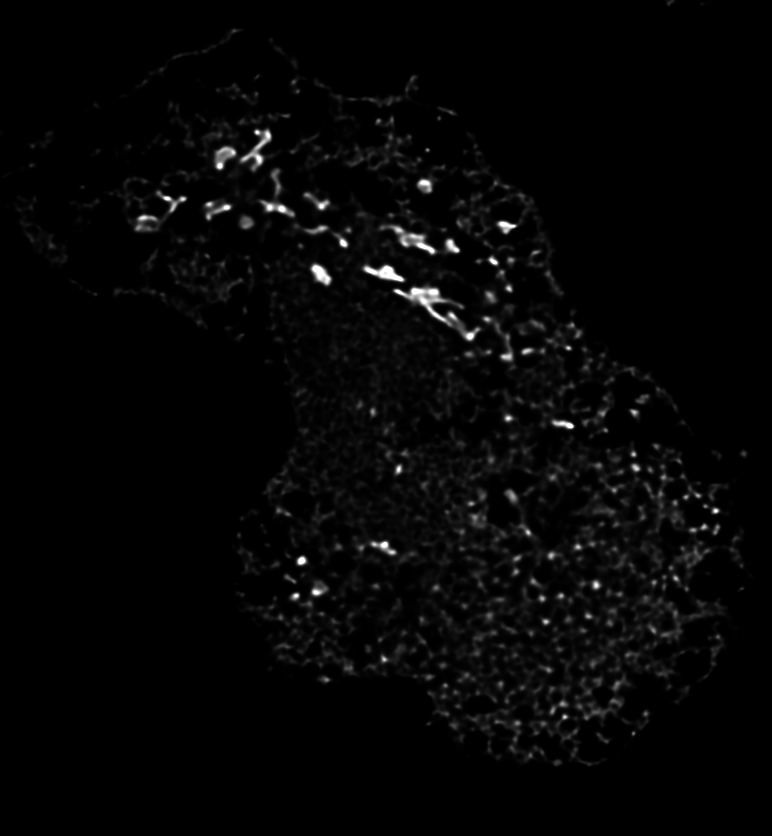

Supplement: Supplementary file 5 — Source data Fig. 4 [file 44321_2026_453_MOESM5_ESM.zip › Figure 4/4C/GCSFD mitotracker.tif]

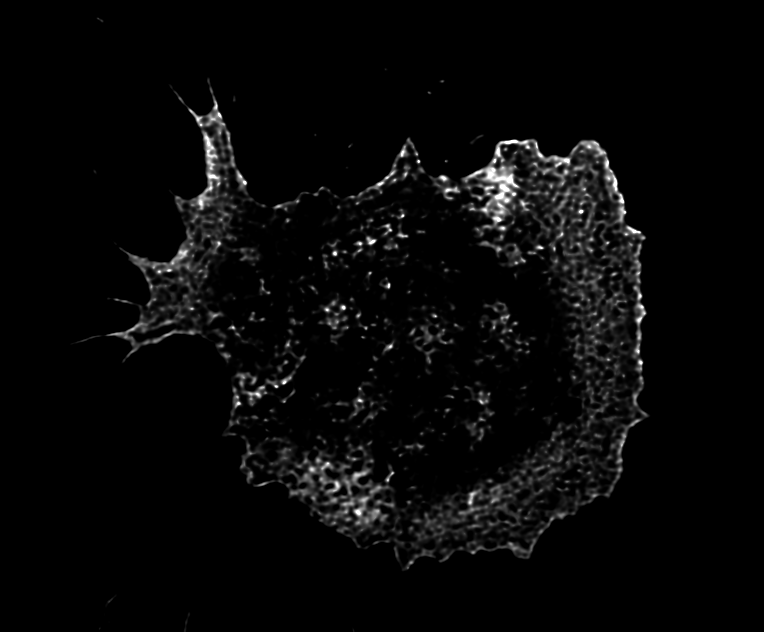

Supplement: Supplementary file 7 — Source data Fig. 6 [file 44321_2026_453_MOESM7_ESM.zip › Figure 6/6B/HC SkQ1 phalloidin.tif]

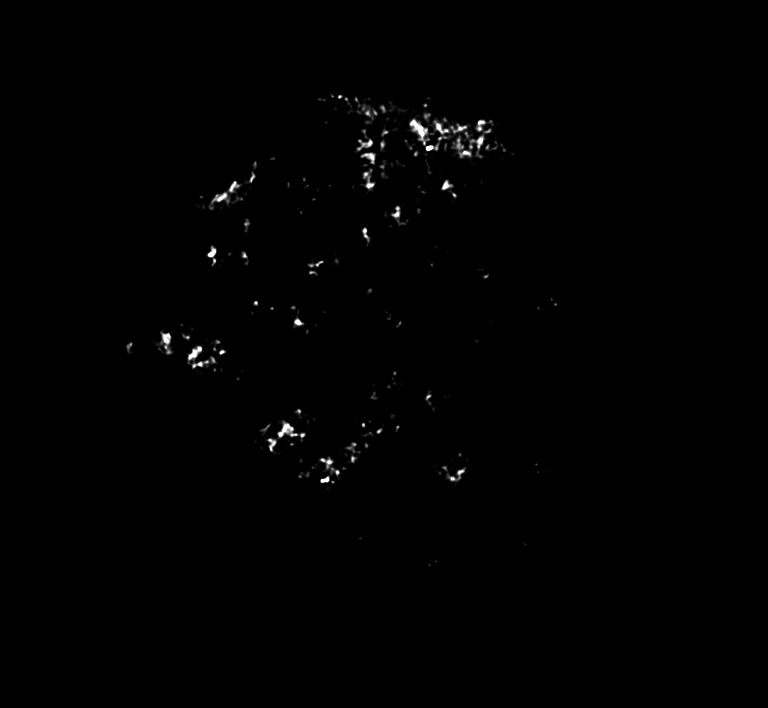

Supplement: Supplementary file 7 — Source data Fig. 6 [file 44321_2026_453_MOESM7_ESM.zip › Figure 6/6B/sepsis SkQ1 phalloidin.tif]
