# Supplementary material for: Mitochondrial activity promotes neutrophil degranulation and endothelial dysfunction in systemic infections
Source: EMBO Mol Med. 2026 May 27;18(7):2691–722. doi: 10.1038/s44321-026-00453-1 (PMC13365472; doi:10.1038/s44321-026-00453-1)
Supplement: Supplementary file 8 — Expanded View Figures [file 44321_2026_453_MOESM8_ESM.pdf]

Expanded View Figures

Figure EV1. Additional analyses of endothelial permeability and glycocalyx shedding.

(**A**) Fold change of syndecan-4 in GEnC culture supernatants following co-culture with peripheral blood neutrophils for 5 h, with or without treatment with TNFα (2 ng/mL), $n = 3$. (**B**) Percentage of GEnC death following culture with untreated, TNFα (2 ng/mL) and A23187 (2.5 μM)-treated neutrophils for 5 h, $n = 3$. (**C**) TEER cell index at 48 h following treatment of HUVECs with NE (0.10–5.00 μg/mL) and positive control (Dengue virus NS1 protein; 5.00 μg/mL) and compared to baseline, $n = 3$. (**D**). TEER cell index at 48 h following treatment of HUVECs with MPO (0.02–2.00 μg/mL) and positive control (Dengue virus NS1 protein; 5.00 μg/mL) and compared to baseline, $n = 3$. (**E**) Representative phase contrast images of HUVECs treated with neutrophil condition media (NCM) for 5 h followed by incubation with freshly isolated neutrophils for 1 h (PMNs) and measured cell confluency after the treatment, $n = 4$. unstim NCM – NCM obtained by incubating neutrophils for 4 h with DMSO; stim NCM – NCM obtained by incubating neutrophils for 4 h with 2.5 μM A23187; NEi – neutrophil elastase inhibitor BAY-678; CGi – cathepsin G inhibitor I; scale bar 100 μm. Data information: Data are presented as mean ± SD. ns not significant, *$P \leq 0.05$, ***$P \leq 0.001$, ****$P \leq 0.0001$, assessed using two-way ANOVA with Tukey's post-hoc test (**A**) and one-way ANOVA with Dunnett's (**B–D**) and Tukey's post-hoc tests (**E**).

 © The Author(s)

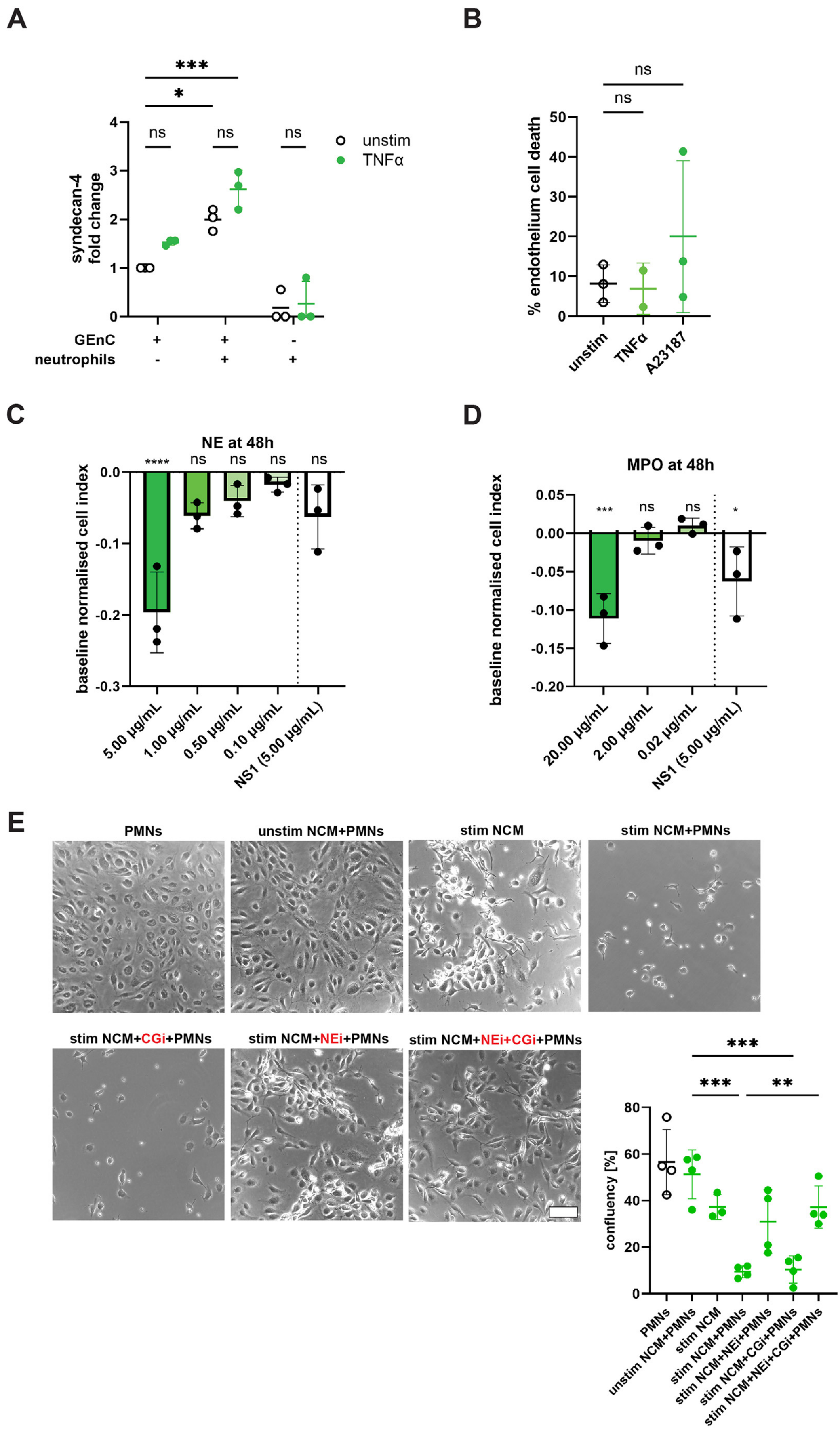
A
B
C
D
E
syndecan-4 fold change
unstim
TNFα
GEnC
neutrophils
ns
*
***
% endothelium cell death
unstim
TNFα
A23187
NE at 48h
baseline normalised cell index
5.00 µg/mL
1.00 µg/mL
0.50 µg/mL
0.10 µg/mL
NS1 (5.00 µg/mL)
MPO at 48h
20.00 µg/mL
2.00 µg/mL
0.02 µg/mL
PMNs
unstim NCM+PMNs
stim NCM
stim NCM+PMNs
stim NCM+CGi+PMNs
stim NCM+NEi+PMNs
stim NCM+NEi+CGi+PMNs
confluency [%]

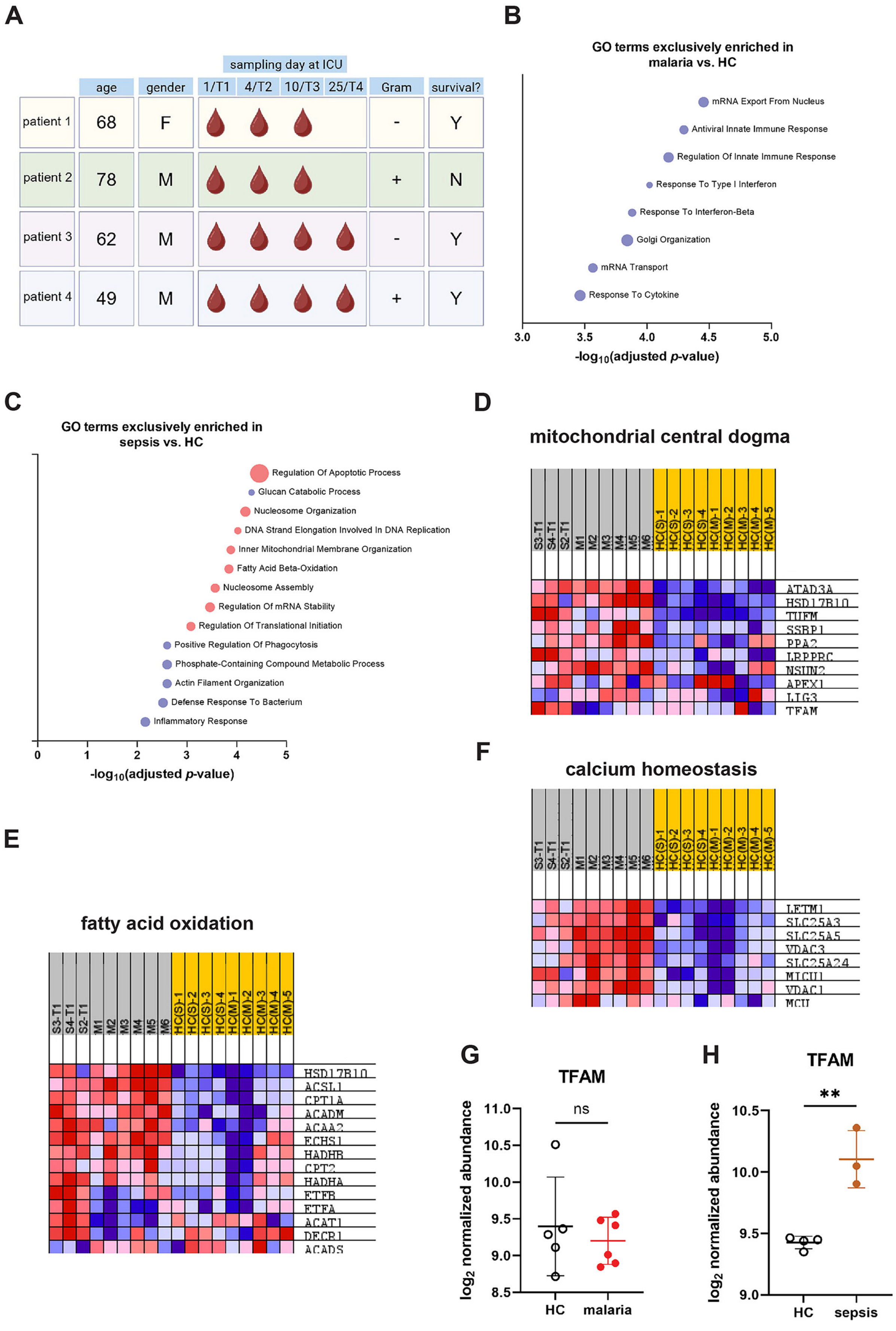
A
sampling day at ICU
age
gender
1/T1
4/T2
10/T3
25/T4
Gram
survival?
patient 1
68
F
-
Y
patient 2
78
M
+
N
patient 3
62
M
-
Y
patient 4
49
M
+
Y
B
GO terms exclusively enriched in malaria vs. HC
mRNA Export From Nucleus
Antiviral Innate Immune Response
Regulation Of Innate Immune Response
Response To Type I Interferon
Response To Interferon-Beta
Golgi Organization
mRNA Transport
Response To Cytokine
3.0
3.5
4.0
4.5
5.0
-log10(adjusted p-value)
C
GO terms exclusively enriched in sepsis vs. HC
Regulation Of Apoptotic Process
Glucan Catabolic Process
Nucleosome Organization
DNA Strand Elongation Involved In DNA Replication
Inner Mitochondrial Membrane Organization
Fatty Acid Beta-Oxidation
Nucleosome Assembly
Regulation Of mRNA Stability
Regulation Of Translational Initiation
Positive Regulation Of Phagocytosis
Phosphate-Containing Compound Metabolic Process
Actin Filament Organization
Defense Response To Bacterium
Inflammatory Response
0
1
2
3
4
5
-log10(adjusted p-value)
D
mitochondrial central dogma
S3-T1
S4-T1
S2-T1
M1
M2
M3
M4
M5
M6
HC(S)-1
HC(S)-2
HC(S)-3
HC(S)-4
HC(M)-1
HC(M)-2
HC(M)-3
HC(M)-4
HC(M)-5
ATAD3A
HSD17B10
TUFM
SSBP1
PPA2
LRPPRC
NSUN2
APEX1
LIG3
TFAM
E
fatty acid oxidation
HSD17B10
ACSL1
CPT1A
ACADM
ACAA2
ECHS1
HADHB
CPT2
HADHA
ETFB
ETFA
ACAT1
DECR1
ACADS
F
calcium homeostasis
LETM1
SLC25A3
SLC25A5
VDAC3
SLC25A24
MICU1
VDAC1
MCU
G
TFAM
ns
log2 normalized abundance
11.0
10.5
10.0
9.5
9.0
8.5
HC
malaria
H
TFAM
**
log2 normalized abundance
10.5
10.0
9.5
9.0
HC
sepsis

 © The Author(s)

◀ **Figure EV2. Additional bioinformatics analysis.**

(**A**) Detailed schematic of blood collection from sepsis patients. (**B**, **C**) Gene Set Enrichment Analysis (GSEA) results showing GO terms exclusively enriched in malaria vs. HC (**B**) and sepsis vs. HC (**C**) datasets. (**D–F**) GSEA results showing enriched MitoCarta terms. (**G**, **H**) Individual normalized abundances of TFAM peptide in malaria vs. HC (**G**) and sepsis vs. HC (**H**) neutrophils. Samples from malaria patients were collected at admission; these patients are designated M1–M6. Samples from sepsis patients were collected on days 1, 4, 10 and 25 following admission to the intensive care unit and are designated T1–T4 for each patient (patients S1–S4). Healthy controls on (**D–F**) are indicated HC(S) for sepsis dataset and HC(M) for malaria dataset. Data information: Data are presented as mean ± SD. ns not significant, $^{**}P \leq 0.01$, assessed using hypergeometric test with *P* values adjusted using the Benjamini–Hochberg method (**B**, **C**) or unpaired *t* test (**G**, **H**).

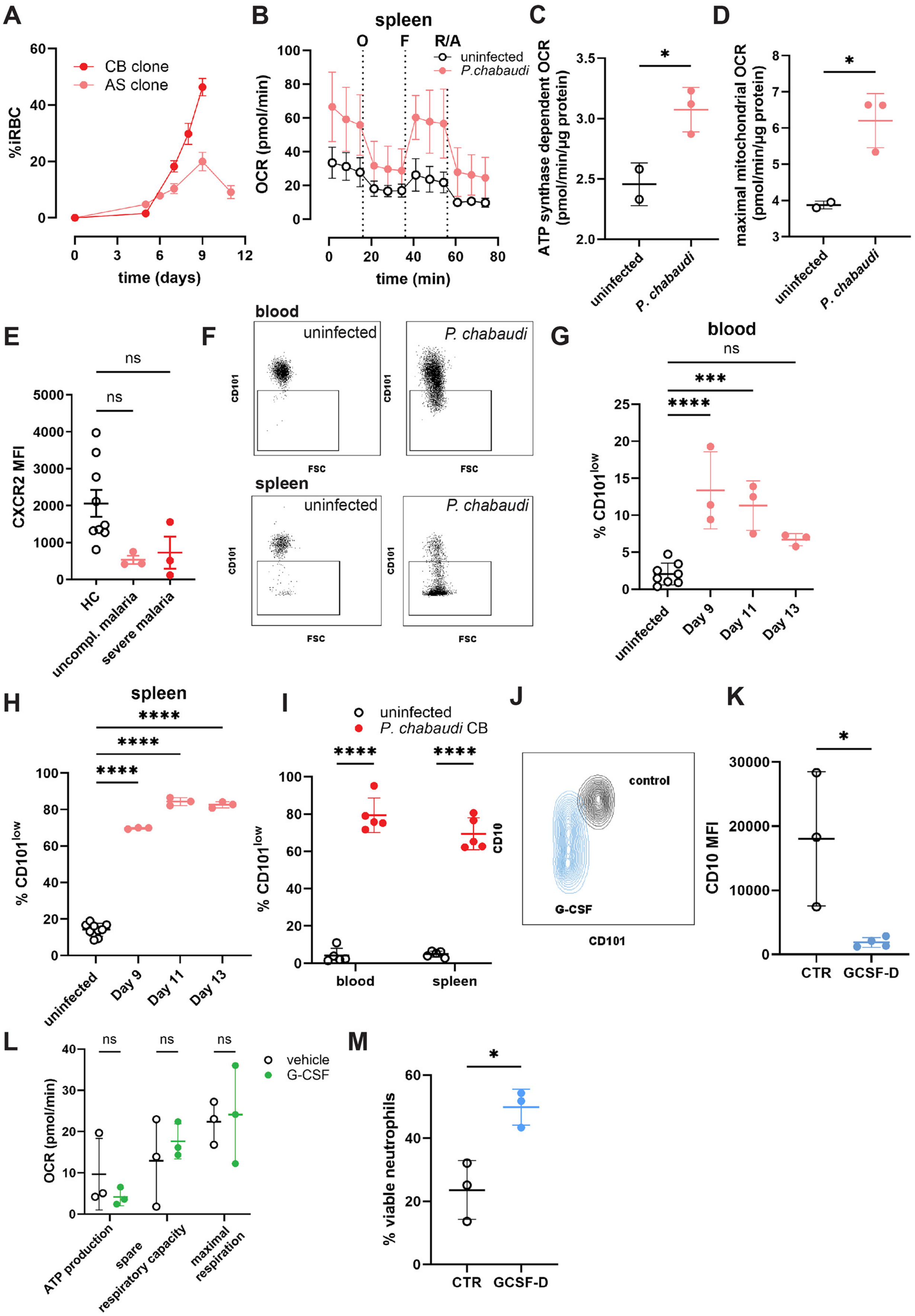
A
CB clone
AS clone
%iRBC
time (days)
B
spleen
O F R/A
uninfected
P.chabaudi
OCR (pmol/min)
time (min)
C
ATP synthase dependent OCR (pmol/min/µg protein)
uninfected
P. chabaudi
D
maximal mitochondrial OCR (pmol/min/µg protein)
uninfected
P. chabaudi
E
CXCR2 MFI
ns
HC
uncompl. malaria
severe malaria
F
blood
uninfected
P. chabaudi
spleen
CD101
FSC
G
blood
ns
% CD101low
uninfected
Day 9
Day 11
Day 13
H
spleen
% CD101low
I
uninfected
P. chabaudi CB
blood
spleen
J
control
G-CSF
CD10
CD101
K
CD10 MFI
CTR
GCSF-D
L
OCR (pmol/min)
vehicle
G-CSF
ATP production
spare respiratory capacity
maximal respiration
M
% viable neutrophils
CTR
GCSF-D

 © The Author(s)

Figure EV3. Additional analyses of mitochondrial function in malaria-elicited and GCSF-D neutrophils.

(**A**) Percentage of infected red blood cells with *P. chabaudi* clone AS ($n = 3$) and CB ($n = 5$) in the circulation of mice. (**B**) OCR of spleen neutrophils of *P. chabaudi* clone AS-infected mice ($n = 3$). Seahorse port injections are indicated as: O – oligomycin (1.5 µM), F – FCCP (400 nM), R/A – rotenone (1 µM) and antimycin A (1 µM). (**C**) ATP synthase dependent OCR of splenic neutrophils isolated from *P. chabaudi* clone AS-infected mice on day 9 post-infection ($n = 3$). (**D**) Maximal mitochondrial OCR of splenic neutrophils isolated from *P. chabaudi* clone AS-infected mice on day 9 post-infection ($n = 3$). (**E**) Median fluorescence intensity of CXCR2 on peripheral blood neutrophils from HC, and patients with uncomplicated and severe malaria, $n = 9$ (HC), 3 (uncomplicated malaria), 3 (severe malaria). (**F**) Representative flow cytometry plots showing CD101 expression on peripheral blood and splenic neutrophil from mice at day 9 post-infection with *P. chabaudi* clone AS. (**G**) Percentage of $CD101^{low}$ peripheral blood neutrophils from *P. chabaudi* clone AS-infected mice at days 9, 11 and 13 post-infection. $n = 8$ (uninfected mice), 3 (infected mice). (**H**) Percentage of $CD101^{low}$ splenic neutrophils from *P. chabaudi* clone AS-infected mice at days 9, 11 and 13 post-infection. $n = 8$ (uninfected mice), 3 (infected mice). (**I**). Percentage of $CD101^{low}$ neutrophils in peripheral blood and spleen from mice infected with *P. chabaudi* clone CB at day 9 post-infection, $n = 5$. (**J**) Representative flow cytometry contour plot for CD10 and CD101 expression on peripheral blood neutrophils derived from control (CTR) and GCSF-treated (GCSF-D) donors. (**K**) Median fluorescence intensity of CD10 on peripheral blood neutrophils from CTR and GCSF-D donors, $n = 3$ (CTR), 4 (GCSF-D). (**L**) ATP production, spare respiratory capacity, and maximal respiration of CTR neutrophils treated ex vivo with 10 ng/mL G-CSF for 30 min, $n = 4$. (**M**) Percentage of viable CTR and GCSF-D neutrophils incubated overnight in complete media, $n = 3$. Data information: Data are presented as mean ± SD. ns – not significant, $*P \le 0.05$, $***P \le 0.001$, $****P \le 0.0001$, assessed using unpaired *t* test (**C**, **D**, **K**, **M**), one-way ANOVA with Dunnett's post-hoc test (**E**, **G**, **H**), and two-way ANOVA with Šidák's post-hoc test (**I**, **L**).

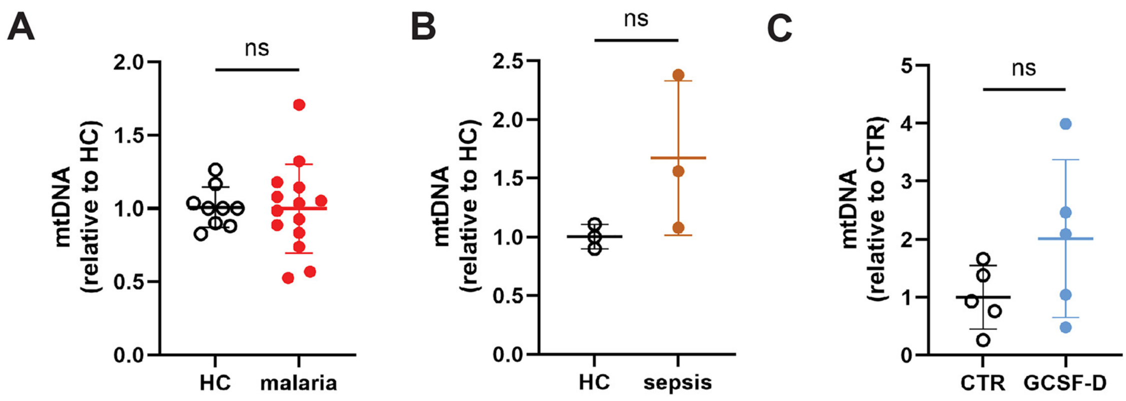

Figure EV4. Additional analyses of mitochondria in neutrophils.

(**A**) Relative mtDNA content in neutrophils from malaria patients ($n = 14$) compared to HC ($n = 9$). (**B**). Relative mtDNA content in neutrophils from sepsis patients ($n = 3$) compared to HC ($n = 3$). (**C**) Relative mtDNA content in neutrophils from GCSF-D donors ($n = 5$) compared to CTR neutrophils ($n = 5$). Data information: Data are presented as mean ± SD. ns – not significant, assessed using unpaired *t* test.

 © The Author(s)

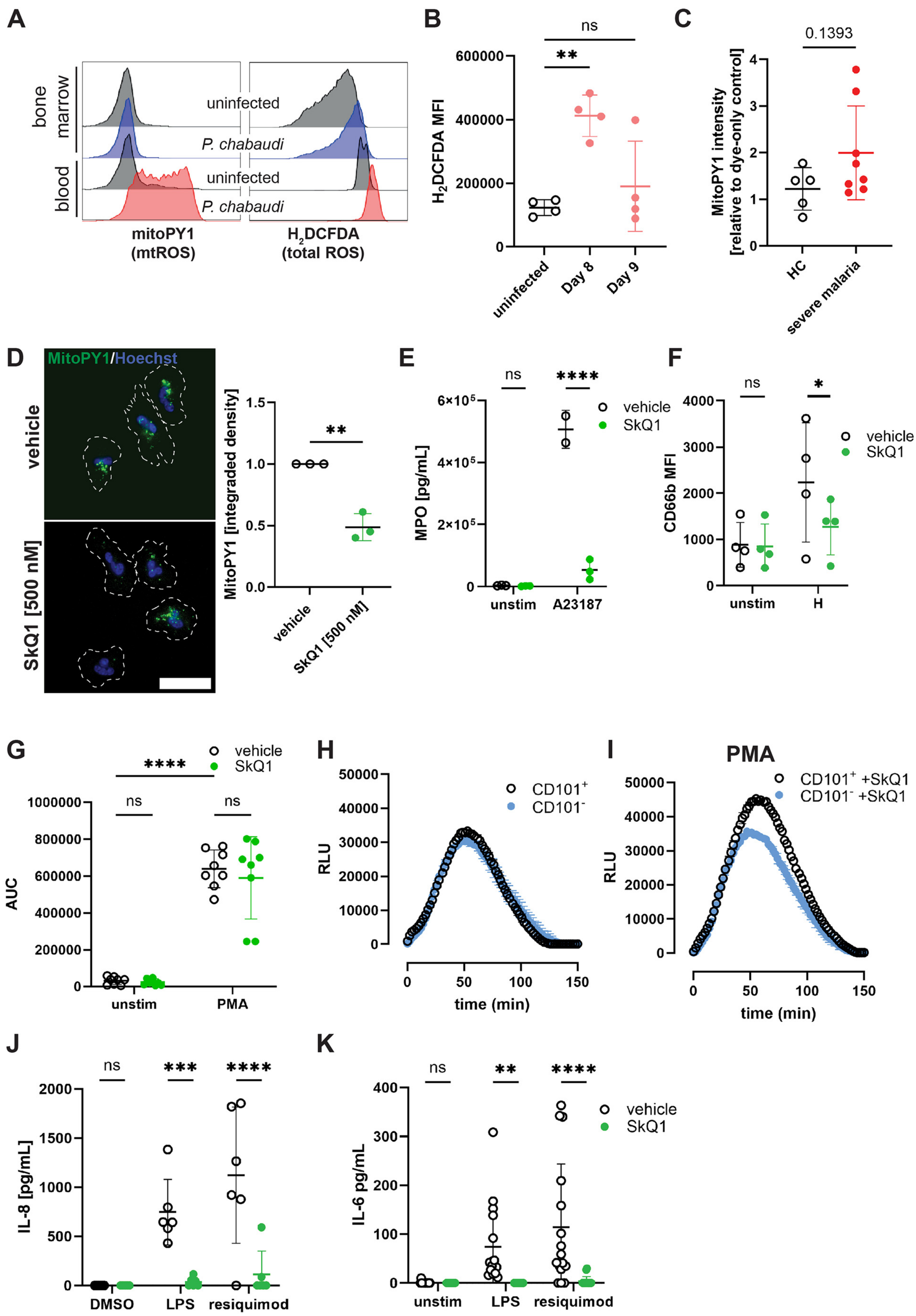
A
bone marrow
blood
uninfected
P. chabaudi
uninfected
P. chabaudi
mitoPY1 (mtROS)
H2DCFDA (total ROS)
B
ns
**
H2DCFDA MFI
uninfected
Day 8
Day 9
C
0.1393
MitoPY1 intensity [relative to dye-only control]
HC
severe malaria
D
MitoPY1/Hoechst
vehicle
SkQ1 [500 nM]
**
MitoPY1 [integraded density]
E
ns
****
vehicle
SkQ1
MPO [pg/mL]
unstim
A23187
F
ns
*
vehicle
SkQ1
CD66b MFI
unstim
H
G
****
ns
ns
vehicle
SkQ1
AUC
unstim
PMA
H
CD101+
CD101-
RLU
time (min)
I
PMA
CD101+ +SkQ1
CD101- +SkQ1
RLU
time (min)
J
ns
***
****
IL-8 [pg/mL]
DMSO
LPS
resiquimod
K
ns
**
****
vehicle
SkQ1
IL-6 pg/mL
unstim
LPS
resiquimod

Figure EV5. mtROS regulates neutrophil degranulation, NOX2-dependent oxidative burst and cytokine release.

(**A**) Representative FACS histograms showing mtROS (MitoPY1) and total ROS (H_2DCFDA) profiles of blood and bone marrow neutrophils from uninfected and *P. chabaudi* clone AS-infected mouse. (**B**) Median fluorescence intensity of H_2DCFDA in neutrophils from uninfected and *P. chabaudi* clone AS-infected mice at day 8 and 9 post-infection, $n = 4$. (**C**) Fluorescence intensity of MitoPY1 in neutrophils from HC ($n = 5$) and cerebral malaria patients ($n = 8$) relative to dye-only control. (**D**) Left: Representative widefield fluorescence images of CTR neutrophils stained with MitoPY1 and Hoechst, pre-incubated with vehicle (DMSO) or SkQ1 (500 nM), scale bar 10 µm. Dashed borders outline cell boundaries. Right: MitoPY1 integrated density, $n = 3$. (**E**). Myeloperoxidase (MPO) levels in supernatants of CTR neutrophils stimulated with calcium ionophore A23187 (2.5 µM), pre-incubated with vehicle (DMSO) and SkQ1 (500 nM), $n = 3$. (**F**) Median fluorescence intensity of CD66b on peripheral blood neutrophils from CTR donors stimulated with heme (30 µM), $n = 4$. (**G**). Quantification of oxidative burst (as area under curve, AUC) of CTR neutrophils stimulated with PMA (100 nM), pre-incubated with vehicle (DMSO) and SkQ1 (500 nM) $n = 8$. (**H**) Representative luminol-based measurement of ROS production in FACS-sorted mouse $CD101^-$ and $CD101^+$ neutrophils stimulated with PMA (100 nM). (**I**) Representative luminol-based measurement of ROS production in FACS-sorted mouse $CD101^-$ and $CD101^+$ neutrophils stimulated with PMA (100 nM) and pre-stimulated with SkQ1 (500 nM). (**J**) Interleukin 8 (IL-8) levels in supernatants of CTR neutrophils stimulated overnight with LPS (100 ng/mL; $n = 6$) and resiquimod (5 µM; $n = 5$), (**K**) Interleukin 6 (IL-6) levels in supernatants of CTR neutrophils stimulated overnight with LPS (100 ng/mL) and resiquimod 5 µM), $n = 13$ (LPS), 15 (resiquimod). Data information: Data are presented as mean ± SD. ns not significant, *$P \le 0.05$, **$P \le 0.01$, ***$P \le 0.001$, ****$P \le 0.0001$, assessed using one-way ANOVA with Dunnett's post-hoc test (**B**), unpaired *t* test (**C**, **D**) and two-way ANOVA with Tukey's (**E–G**, **J**, **K**).

© The Author(s)
