# Supplementary material for: Mitochondrial activity promotes neutrophil degranulation and endothelial dysfunction in systemic infections
Source: EMBO Mol Med. 2026 May 27;18(7):2691–722. doi: 10.1038/s44321-026-00453-1 (PMC13365472; doi:10.1038/s44321-026-00453-1)
Supplement: Supplementary file 2 — Peer Review File [file 44321_2026_453_MOESM2_ESM.pdf]

EMBO Molecular Medicine

# Mitochondrial activity promotes neutrophil degranulation and endothelial dysfunction in systemic infections

Przemyslaw Zakrzewski, Christopher Rice, Claire Naveh, Isaac Dowell, Kathryn Fleming, Aravind Ramesh, Rachel Jones, Pedro Moura, Drinalda Cela, Sarah Groves, Stephanie Fletcher-Jones, Yohance Victory, Mainga Bhima, Stefan Ebmeier, Laura Carey, Matthew Butler, Simon Satchell, Ase Berg, Nadia Palolite, James Nyirenda, Watipenge Nyasulu, Isabel Zgambo, Charalampos Attipa, Linda Wooldridge, Andrew Davidson, Aubrey Cunnington, Christopher Moxon, and Borko Amulic

*Corresponding author(s): Borko Amulic (borko.amulic@bristol.ac.uk)*

## Review Timeline:

| | |
|---|---|
| Submission Date: | 10th Oct 25 |
| Editorial Decision: | 28th Nov 25 |
| Revision Received: | 1st Apr 26 |
| Editorial Decision: | 24th Apr 26 |
| Revision Received: | 8th May 26 |
| Accepted: | 11th May 26 |

*Editor: Zeljko Durdevic*

## Transaction Report:

(Note: With the exception of the correction of typographical or spelling errors that could be a source of ambiguity, letters and reports are not edited. Depending on transfer agreements, referee reports obtained elsewhere may or may not be included in this compilation. Referee reports are anonymous unless the Referee chooses to sign their reports.)

28th Nov 2025

Dear Dr. Amulic,

Thank you for the submission of your manuscript to EMBO Molecular Medicine, and please accept my apologies for the unusual delay in getting back to you. We have now received feedback from two of the three reviewers who agreed to evaluate your manuscript. As the referee #2 will unfortunately not be able to return his/her report in a timely manner, we prefer to make a decision now in order to avoid further delay in the process. Should referee #2 provide a report, we will send it to you, with the understanding that we will not ask for an additional revision.

As you will see from their reports pasted below, both referees recognize potential interest of the manuscript but also raise important concerns that should be addressed in a major revision. If you would like to discuss further the points raised by the referees, I am available to do so via email or video. Let me know if you are interested in this option.

Further consideration of a revision that addresses reviewers' concerns in full will entail a second round of review. EMBO Molecular Medicine encourages a single round of revision only and therefore, acceptance or rejection of the manuscript will depend on the completeness of your responses included in the next, final version of the manuscript. For this reason, and to save you from any frustrations in the end, I would strongly advise against returning an incomplete revision.

We would welcome the submission of a revised version within three months for further consideration. Please let us know if you require longer to complete the revision.

I look forward to receiving your revised manuscript.

Yours sincerely,

Zeljko Durdevic

Zeljko Durdevic
Senior Editor
EMBO Molecular Medicine

*****
When preparing your revised manuscript, please refer to our guidelines: https://link.springer.com/journal/44321/submission-guidelines#cms-Revised-submissions. We perform an initial quality control of all revised manuscripts before re-review; failure to include requested items will delay the evaluation of your revision.

We require:

1) A .docx formatted version of the manuscript text (including legends for main figures, EV figures and tables). Please make sure that the changes are highlighted to be clearly visible.

2) Individual production quality figure files as .eps, .tif, .jpg (one file per figure). For guidance, download the 'Figure Guide PDF': https://media.springernature.com/original/springer-cms/rest/v1/content/27825798/data/v1.

3) A .docx formatted letter INCLUDING the reviewers' reports and your detailed point-by-point responses to their comments. As part of the EMBO Press transparent editorial process, the point-by-point response is part of the Review Process File (RPF), which will be published alongside your paper.

4) A complete author checklist, which you can download from our author guidelines. Please insert information in the checklist that is also reflected in the manuscript. The completed author checklist will also be part of the RPF.

5) Please note that all corresponding authors are required to supply an ORCID ID for their name upon submission of a revised

manuscript.

6) It is mandatory to include a 'Data Availability' section after the Materials and Methods. Before submitting your revision, primary datasets produced in this study need to be deposited in an appropriate public database, and the accession numbers and database listed under 'Data Availability'. Please remember to provide a reviewer password if the datasets are not yet public.

In case you have no data that requires deposition in a public database, please state so in this section. Note that the Data Availability Section is restricted to new primary data that are part of this study.

7) For data quantification: please specify the name of the statistical test used to generate error bars and P values, the number (n) of independent experiments (specify technical or biological replicates) underlying each data point and the test used to calculate p-values in each figure legend. The figure legends should contain a basic description of n, P and the test applied. Graphs must include a description of the bars and the error bars (s.d., s.e.m.).

8) At EMBO Press we ask authors to provide source data for the main manuscript figures. You will receive a separate email with instructions for providing source data with your revised manuscript, including how to upload and organize the files.

9) Our journal encourages inclusion of *data citations in the reference list* to directly cite datasets that were re-used and obtained from public databases. Data citations in the article text are distinct from normal bibliographical citations and should directly link to the database records from which the data can be accessed. In the main text, data citations are formatted as follows: "Data ref: Smith et al, 2001" or "Data ref: NCBI Sequence Read Archive PRJNA342805, 2017". In the Reference list, data citations must be labeled with "[DATASET]". A data reference must provide the database name, accession number/identifiers and a resolvable link to the landing page from which the data can be accessed at the end of the reference.

10) We replaced Supplementary Information with Expanded View (EV) Figures and Tables that are collapsible/expandable online. A maximum of 5 EV Figures can be typeset. EV Figures should be cited as 'Figure EV1, Figure EV2" etc... in the text and their respective legends should be included in the main text after the legends of regular figures.

- For the figures that you do NOT wish to display as Expanded View figures, they should be bundled together with their legends in a single PDF file called *Appendix*, which should start with a short Table of Content. Appendix figures should be referred to in the main text as: "Appendix Figure S1, Appendix Figure S2" etc.

- Additional Tables/Datasets should be labeled and referred to as Table EV1, Dataset EV1, etc. Legends have to be provided in a separate tab in case of .xls files. Alternatively, the legend can be supplied as a separate text file (README) and zipped together with the Table/Dataset file.

11) The paper explained: EMBO Molecular Medicine articles are accompanied by a summary of the articles to emphasize the major findings in the paper and their medical implications for the non-specialist reader. Please provide a draft summary of your article highlighting

- the medical issue you are addressing,

- the results obtained and

- their clinical impact.

This may be edited to ensure that readers understand the significance and context of the research. Please refer to any of our published articles for an example.

12) Author contributions: You will be asked to provide CRediT (Contributor Role Taxonomy) terms in the submission system. These replace a narrative author contribution section in the manuscript.

13) A Conflict of Interest statement should be provided in the main text.

14) Every published paper includes a 'Synopsis' to further enhance discoverability. Synopses are displayed on the journal webpage and are freely accessible to all readers. They include a short stand first (maximum of 300 characters, including space) as well as 2-5 one-sentences bullet points that summarizes the paper. Please write the bullet points to summarize the key NEW findings. They should be designed to be complementary to the abstract - i.e. not repeat the same text. We encourage inclusion of key acronyms and quantitative information (maximum of 30 words / bullet point). Please use the passive voice. Please attach these in a separate file or send them by email, we will incorporate them accordingly.

Please also suggest a striking image or visual abstract to illustrate your article as a PNG file 550 px wide x 300-600 px high.

15) Include a Reagents and Tools Table as part of the Methods section, which can be downloaded from our author guidelines.

EMBO Molecular Medicine has a "scooping protection" policy, whereby similar findings that are published by others during review or revision are not a criterion for rejection. Should you decide to submit a revised version, I do ask that you get in touch after three months if you have not completed it, to update us on the status.

***** Reviewer's comments *****

Referee #1 (Comments on Novelty/Model System for Author):

When feasible the authors tried to use relevant models to study their pathology of interest, here severe malaria disease. A strong human-based study was performed with blood samples from various patients and healthy donors (medical and ethical agreements OK)as well as rodent models when physiology could not be studied in humpan samples.
The statistical tests when fairly used and are all appropriated to the classical use of tests in such settings.

Referee #1 (Remarks for Author):

Review on the study entitled « Mitochondrial activity promotes neutrophil degranulation and endothelial dysfunction in malaria infection » from Zakrzewski et al.,
In this study, the authors adress an interesting effect of neutrophil degranulation on the endothelial permeability that occurs in various pathological contextes such as severe infections/inflammation. They show that 1/ neutrophil degranulation contibutes via its primary and secondary granules to glycocalyx degradation and subsequent endothelial dysfunction and 2/ that this function is strongly tuned by mitochondrial ROS mostly produced by immature neutrophils.
This is an important study that combines both in vitro and patient samples.
There are some points that would deserve to be clarified in order to support the author' conclusions :
1/ While the authors show that NE but not PR3 can affect the endothelial response, the 3rd and fourth granule proteases, namely Cathepsin G and NSP4 remain not adressed. If the authors aim to validate that neutrophil proteases that are degranulated will affect the endothelial barrier resposne, having those two proteases included would help discriminating about their individual role or absence of role.
2/ A point to adress is the contribution of the NE (or other proteases in the studied process). Indeed, the point of the authors that mtROS-driven Protease release and the subsequent protease-driven glyocalyx alterations and endothelial permeabilization is aonly partly covered with the use SkQ1 (mtROS targeting). The use of NE or even Cathepsin C (indirectly targeting neutrophil NE/CathG/Pr3 proteases) inhibitors that exist and work perfectly in these settings would also give to the authors the final validation of the link between mtROS-protease release and activated protease-induced glycocalyx degradation.
3/ If the explanation that SkQ1-inhibited mtROS lead to cortical actin stabilization and inhibition of the release of granules, a control to include should be jasplakinolides (strong actin stabilizers) to determine its effect on the studied processes.
4/ Calcium signalling also links PAD2/4-driven NETOsis. Have the authors checked if the studied process was due to degranulation of PAD2/4-driven DNA decondensation and protease release ? PAD4 inhibitors (Clamidine or GSK384) could easily help doing this control given the strong expertize of the group in this field or research.

Referee #3 (Remarks for Author):

Zakrzewski et al in their study entitled 'Mitochondrial activity promotes neutrophil degranulation and endothelial dysfunction in systemic infections' have investigated the regulation of neutrophil activity and the implications in endothelial function using models of systemic infection.

The research question is timely and the authors have presented strong evidence to support their research hypothesis and a clear list of study limitations.

However, clarifications on specific points would be required to strengthen the impact of the study.

- The authors have shown that neutrophil degranulation enhances endothelial permeability and glycocalyx shedding. It would provide significant mechanistic insight if the authors can determine whether neutrophil adhesion to the endothelial layer is affected in their experimental model as a result of glycocalyx shedding.

- In addition, this study presents a proteomic analysis dataset that demonstrates enrichment of mitochondria in neutrophils

isolated from patients with severe malaria and sepsis, suggesting metabolic rewiring and potential for OXPHOS utilisation. These findings would need further discussion regarding their fit in the current literature and clearer description of the novel elements of this study.

- Did the immunophenotyping analysis of immature neutrophils include any assessment of their survival and functions beyond ROS release?

**EMM-2025-22759-V2**

**Response to reviewers**

***** Reviewer's comments *****

Referee #1 (Comments on Novelty/Model System for Author):

When feasible the authors tried to use relevant models to study their pathology of interest, here severe malaria disease. A strong human-based study was performed with blood samples from various patients and healthy donors (medical and ethical agreements OK)as well as rodent models when physiology could not be studied in humpan samples.
The statistical tests when fairly used and are all appropriated to the classical use of tests in such settings.

Referee #1 (Remarks for Author):

***Review on the study entitled « Mitochondrial activity promotes neutrophil degranulation and endothelial dysfunction in malaria infection » from Zakrzewski et al.,***
***In this study, the authors adress an interesting effect of neutrophil degranulation on the endothelial permeability that occurs in various pathological contextes such as severe infections/inflammation. They show that 1/ neutrophil degranulation contibutes via its primary and secondary granules to glycocalyx degradation and subsequent endothelial dysfunction and 2/ that this function is strongly tuned by mitochondrial ROS mostly produced by immature neutrophils.***
***This is an important study that combines both in vitro and patient samples.***
***There are some points that would deserve to be clarified in order to support the author' conclusions :***

***1/ While the authors show that NE but not PR3 can affect the endothelial response, the 3rd and fourth granule proteases, namely Cathepsin G and NSP4 remain not adressed. If the authors aim to validate that neutrophil proteases that are degranulated will affect the endothelial barrier resposne, having those two proteases included would help discriminating about their individual role or absence of role.***

We thank the reviewer for this suggestion to investigate the two remaining primary granule proteins. We tested the role of cathepsin G in endothelial disruption in an additional experiment that used microscopy to examine the integrity of the endothelial monolayer. We show that treatment of a confluent HUVEC monolayer with neutrophil conditioned medium (NCM), containing exocytosed granule components, leads to a partial reduction in endothelial confluency, which is further exacerbated with addition of intact neutrophils. The neutrophil elastase inhibitor (NEi) had a protective effect that partially reversed this disruption, consistent with our original findings. The cathepsin G inhibitor (CGi) alone had minimal effect on endothelial confluency. However, a greater degree of protection was observed when NEi and CGi were combined, restoring confluency towards levels seen with NCM only. This is now included in Fig. EV1E.

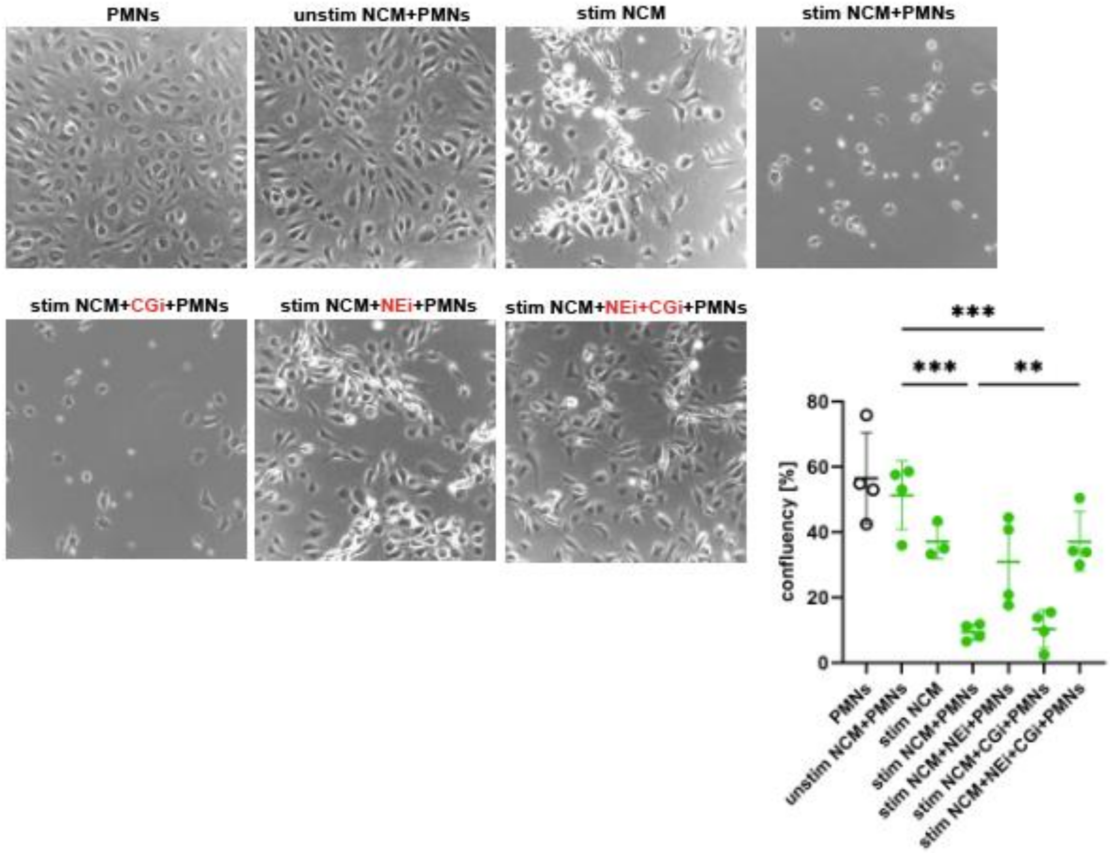


NSP4 is the most recently characterised primary granule protease (1) and for this reason there are few reagents available to investigate it. Unfortunately, we were not able to test the role of NSP4 since there were no commercially available reagents to inhibit it, and we couldn't acquire recombinant protein. We have pointed out this caveat in the discussion (Limitations to our study, page 10, lines 18-21): "*we focused on the endothelial-disruptive effects of only four neutrophil granule proteases and acknowledge that additional modulation may come from untested ones, such as neutrophil serine protease 4 and metalloproteinases 8 and 9*."

1. Perera NC, Schilling O, Kittel H, Back W, Kremmer E, Jenne DE. NSP4, an elastase-related protease in human neutrophils with arginine specificity. Proc Natl Acad Sci U S A. 2012 Apr 17;109(16):6229-34.

**2/ A point to adress is the contribution of the NE (or other proteases in the studied process). Indeed, the point of the authors that mtROS-driven Protease release and the subsequent protease-driven glyocalyx alterations and endothelial permeabilization is aonly partly covered with the use SkQ1 (mtROS targeting). The use of NE or even Cathepsin C (indirectly targeting neutrophil NE/CathG/Pr3 proteases) inhibitors that exist and work perfectly in these settings would also give to the authors the final validation of the link between mtROS-protease release and activated protease-induced glycocalyx degradation.**

We agree with the reviewer and for this reason we included the NE and CG inhibitor experiment described above (Fig. EV1E). We directly observed endothelial barrier

disruption by quantifying loss of confluency. As shown above, inhibition of NE has a significant protective effect on endothelial monolayer integrity.

**3/ If the explanation that SkQ1-inhibited mtROS lead to cortical actin stabilization and inhibition of the release of granules, a control to include should be jasplakinolides (strong actin stabilizers) to determine its effect on the studied processes.**

We agree with the reviewer and in fact this has been previously published. Mitchel et al. (1) showed that jasplakinolide inhibits release of primary granule component MPO. We therefore included this references on page 8, line 21 (reference 58).

To further strengthen the finding of cortical actin destabilisation, we included additional confocal imaging experiments in neutrophils from both control donors and sepsis patients. Fig 6B-C demonstrates that SkQ1 enhances the thickness of actin in the cell periphery, in both mature and immature neutrophils.


1. Mitchell T, Lo A, Logan MR, Lacy P, Eitzen G. Primary granule exocytosis in human neutrophils is regulated by Rac-dependent actin remodeling. Am J Physiol Cell Physiol. 2008 Nov;295(5):C1354-65.


**4/ Calcium signalling also links PAD2/4-driven NETOsis. Have the authors checked if the studied process was due to degranulation of PAD2/4-driven DNA decondensation and protease release ? PAD4 inhibitors (Clamidine or GSK384) could easily help doing this control given the strong expertize of the group in this field or research.**

This is an excellent suggestion – as the reviewer correctly points out, PAD2/4 are strongly calcium dependent and known to be important neutrophil regulators. We tested the PAD4 inhibitor GSK484 for ability to modulate NE release and found no significant effect on degranulation. This is now included as Fig. 6A. We are at this point unable to rule out the involvement of PAD2, as GSK484 specifically targets PAD4 and not PAD2. We have pointed out this caveat in the manuscript on page 10, lines 21-23: "*PAD2 is also activated by calcium ionophores but its role was not tested with the highly selective GSK484 drug*".

**Referee #3 (Remarks for Author):**

**Zakrzewski et al in their study entitled 'Mitochondrial activity promotes neutrophil degranulation and endothelial dysfunction in systemic infections' have investigated the regulation of neutrophil activity and the implications in endothelial function using models of systemic infection.**

**The research question is timely and the authors have presented strong evidence to support their research hypothesis and a clear list of study limitations.**

**However, clarifications on specific points would be required to strengthen the impact of the study.**

**- The authors have shown that neutrophil degranulation enhances endothelial permeability and glycocalyx shedding. It would provide significant mechanistic insight if the authors can determine whether neutrophil adhesion to the endothelial layer is affected in their experimental model as a result of glycocalyx shedding.**

We thank the reviewer for this suggestion, which makes sense in the context of the inflammatory cascade. In order to answer this, we set up an additional imaging-based assay in which we treated a HUVEC monolayer with neutrophil supernatants (stimulated with calcium ionophore) containing degranulation products, followed by addition of intact neutrophils (5 hours later). Interestingly, we observed a strong effect on barrier integrity: whereas neutrophil supernatants alone only partially affected the shape and confluency of the endothelial cells, the subsequent treatment with neutrophils produced a strong retraction and loss of contact between the endothelial cells. This demonstrates that a two-hit process is required for the barrier loss: initial processing by neutrophil serine proteases, followed by a potent direct disruptive effect of the neutrophils themselves. This has been included as Fig. EV1E.

**- In addition, this study presents a proteomic analysis dataset that demonstrates enrichment of mitochondria in neutrophils isolated from patients with severe malaria and sepsis, suggesting metabolic rewiring and potential for OXPHOS utilisation. These findings would need further discussion regarding their fit in the current literature and clearer description of the novel elements of this study.**

We agree with the reviewer that this warrants further discussion. It has been previously demonstrated that neutrophil progenitors transcribe elevated quantities of mitochondrial genes, including OXPHOS. Our study is, to the best of our knowledge, the first proteomic study of neutrophils isolated from patients with infections, furthermore these are patients characterised by severe inflammation. In addition to providing a proteomic resource for the community, we use Seahorse to demonstrate, for the first time, that human infection-elicited neutrophils can actively use mitochondria to generate substantial amounts of ATP, unlike steady state ones. The novelty of our report further comes from the fact that it is not limited to describing changes in mitochondrial abundance – we provide direct functional evidence that mitochondrial ROS regulate degranulation, calcium-dependent oxidative burst and secretion of IL-8 and IL-6.
We have included additional discussion material on page 8, lines 23-33 to explain this, as suggested by the reviewer.

**- Did the immunophenotyping analysis of immature neutrophils include any assessment of their survival and functions beyond ROS release?**

This is a valuable suggestion. We also compared NET formation between steady state neutrophils and GCSF-mobilised immature neutrophils in response to PMA stimulation and observed a reduced rate of NETosis in the immature population. However, as our future manuscript will focus specifically on NETosis, we have chosen not to include these data in the current study.

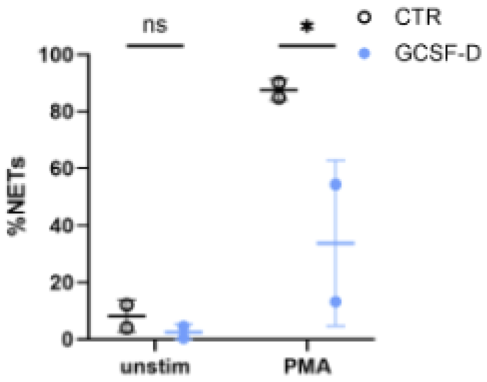


Regarding cell viability, we have now included new data comparing lifespan of control and GCSF-mobilised neutrophils in Fig. EV3M. These results confirm that immature neutrophils exhibit extended lifespan.

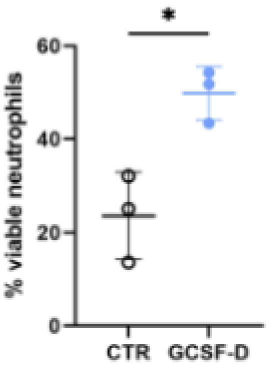

24th Apr 2026

Dear Dr. Amulic,

Thank you for the submission of your revised manuscript to EMBO Molecular Medicine. I am pleased to inform you that we will be able to accept your manuscript pending the following final amendments:

1) Author Checklist: Please add corr. author name, journal and manusciprt number and make sure that all relevant questions are completed.
2) In the main manuscript file, please do the following:
- Please address all comments suggested by our data editors listed below:
o Figure legends:
1. Please note that information related to n is missing in the legends of figures 2F, 3I.
2. Please note that the error bars are not defined in the legend of figure 2F.
3. Please define the annotated p values ****/***/**/* as well as provide the exact p-values for the same in the legend of figure 2F as appropriate.
4. Please note that the exact p values are not provided in the legends of figures 1A-C; 3E, F, G, H, J; 4E, F; 5A-C, D, E, F, G, J; 6C, EV1C, D, E; EV2 H, EV3 C, D, G, H, I, K, M; EV5 D, E, F, G, J, K.
5. Please indicate the statistical test used for data analysis in the legends of figures 2E, F; EV2 B, C.
6. Please note that the dashed borders are not defined in the legend of figure EV5 D. This needs to be rectified.
- The manuscript sections should be in the following order: Title page - Abstract - Introduction - Results - Discussion - Methods - Data Availability - Acknowledgments - Disclosure and Competing Interests Statement - References - Figure Legends - Main Tables with legends - Expanded View Figure Legends.
- There is a callout for Fig. S2, please correct.
- Please rename "Materials and Methods" to "Methods".
- In Methods, provide the antibody dilutions that were used for each antibody.
- In Methods, provide the statement that experiments involving human subjects conformed to the principles set out in the to the WMA Declaration of Helsinki and the Department of Health and Human Services Belmont Report.
- In Methods, add the following paragraph:

Graphics:
(some of the... OR Figure #... OR synopsis) Graphics were created with BioRender.com.
- Indicate in legends exact n and exact p values, not a range, along with the statistical test used. To keep the figures "clear" some authors found providing an Appendix table Sx with all exact p-values preferable. You are welcome to do this if you want to.
- Author contributions: Please remove it from the manuscript and specify author contributions in our submission system. CRediT has replaced the traditional author contributions section because it offers a systematic machine-readable author contributions format that allows for more effective research assessment. You are encouraged to use the free text boxes beneath each contributing author's name to add specific details on the author's contribution. More information is available in our guide to authors:
https://www.embopress.org/page/journal/17574684/authorguide#authorshipguidelines
- In data availability statement please use the following format to report the accession number of your data:

The datasets produced in this study are available in the following databases:
[data type]: [full name of the resource] [accession number/identifier] ([doi or URL or identifiers.org/DATABASE:ACCESSION])

Please check "Author Guidelines" for more information.
https://www.embopress.org/page/journal/17574684/authorguide#availabilityofpublishedmaterial
- Please rename "Bibliography" to "References" and correct the reference citation in the text and reference list. In the text a reference should be cited by author and year of publication. Include a space between a word and the opening parenthesis of the reference that follows. In the reference list, citations should be listed in alphabetical order. Where there are more than 10 authors on a paper, 10 will be listed, followed by "et al.". DOIs should only be used for preprints and datasets that have not been published. Please check "Author Guidelines" for more information.
https://www.embopress.org/page/journal/17574684/authorguide#referencesformat
3) Funding: Please make sure that information about all sources of funding are complete in both our submission system and in the manuscript. Currently, core funding from Wellcome supporting Malawi-Liverpool-Wellcome Research Programme is missing in our submission system. Please correct.
4) The Paper Explained: Please add it to the main manuscript file and rename the subheadings to "Problem", "Results", "Impact".
5) Synopsis:
- Synopsis image: Please resize the image to exactly 550 px-wide x 300 pixels high and upload it as a high-resolution jpeg file.
- Synopsis text: Please remove "The Paper Explained" from the file.
- Please check your synopsis text and image before submission with your revised manuscript. Please be aware that in the proof

stage minor corrections only are allowed (e.g., typos).
6) As part of the EMBO Publications transparent editorial process (see our Editorial at http://embomolmed.embopress.org/content/2/9/329), EMBO Molecular Medicine will publish online a Review Process File (RPF) to accompany accepted manuscripts. This file will be published in conjunction with your paper and will include the anonymous referee reports, your point-by-point response and all pertinent correspondence relating to the manuscript. Let us know if you want to remove or not any figures from it prior to publication. Please note that the Authors checklist will be published at the end of the RPF.
7) Please provide a point-by-point letter INCLUDING my comments as well as the reviewer's reports and your detailed responses (as Word file).

I look forward to reading a new revised version of your manuscript as soon as possible.

Yours sincerely,

Zeljko Durdevic

Zeljko Durdevic
Senior Editor
EMBO Molecular Medicine

*** Instructions to submit your revised manuscript ***

*** PLEASE NOTE *** As part of the EMBO Publications transparent editorial process initiative (see our Editorial at https://www.embopress.org/doi/pdf/10.1002/emmm.201000094), EMBO Molecular Medicine will publish online a Review Process File to accompany accepted manuscripts.

In the event of acceptance, this file will be published in conjunction with your paper and will include the anonymous referee reports, your point-by-point response and all pertinent correspondence relating to the manuscript. If you do NOT want this file to be published, please inform the editorial office at contact@embomolmed.org.

*****

When preparing your revised manuscript, please refer to our guidelines: https://link.springer.com/journal/44321/submission-guidelines#cms-Revised-submissions. We perform an initial quality control of all revised manuscripts before re-review; failure to include requested items will delay the evaluation of your revision.

We require:

1) A .docx formatted version of the manuscript text (including legends for main figures, EV figures and tables). Please make sure that the changes are highlighted to be clearly visible.

2) Individual production quality figure files as .eps, .tif, .jpg (one file per figure). For guidance, download the 'Figure Guide PDF': https://media.springernature.com/original/springer-cms/rest/v1/content/27825798/data/v1.

3) A .docx formatted letter INCLUDING the reviewers' reports and your detailed point-by-point responses to their comments. As part of the EMBO Press transparent editorial process, the point-by-point response is part of the Review Process File (RPF), which will be published alongside your paper.

4) A complete author checklist, which you can download from our author guidelines. Please insert information in the checklist that is also reflected in the manuscript. The completed author checklist will also be part of the RPF.

5) Please note that all corresponding authors are required to supply an ORCID ID for their name upon submission of a revised manuscript.

6) It is mandatory to include a 'Data Availability' section after the Materials and Methods. Before submitting your revision, primary datasets produced in this study need to be deposited in an appropriate public database, and the accession numbers and database listed under 'Data Availability'. Please remember to provide a reviewer password if the datasets are not yet public.

In case you have no data that requires deposition in a public database, please state so in this section. Note that the Data Availability Section is restricted to new primary data that are part of this study.

7) For data quantification: please specify the name of the statistical test used to generate error bars and P values, the number (n) of independent experiments (specify technical or biological replicates) underlying each data point and the test used to calculate p-values in each figure legend. The figure legends should contain a basic description of n, P and the test applied. Graphs must include a description of the bars and the error bars (s.d., s.e.m.).

8) At EMBO Press we ask authors to provide source data for the main manuscript figures. You will receive a separate email with instructions for providing source data with your revised manuscript, including how to upload and organize the files.

9) Our journal encourages inclusion of *data citations in the reference list* to directly cite datasets that were re-used and obtained from public databases. Data citations in the article text are distinct from normal bibliographical citations and should directly link to the database records from which the data can be accessed. In the main text, data citations are formatted as follows: "Data ref: Smith et al, 2001" or "Data ref: NCBI Sequence Read Archive PRJNA342805, 2017". In the Reference list, data citations must be labeled with "[DATASET]". A data reference must provide the database name, accession number/identifiers and a resolvable link to the landing page from which the data can be accessed at the end of the reference.

10) We replaced Supplementary Information with Expanded View (EV) Figures and Tables that are collapsible/expandable online. A maximum of 5 EV Figures can be typeset. EV Figures should be cited as 'Figure EV1, Figure EV2" etc... in the text and their respective legends should be included in the main text after the legends of regular figures.

- For the figures that you do NOT wish to display as Expanded View figures, they should be bundled together with their legends in a single PDF file called *Appendix*, which should start with a short Table of Content. Appendix figures should be referred to in the main text as: "Appendix Figure S1, Appendix Figure S2" etc.

- Additional Tables/Datasets should be labeled and referred to as Table EV1, Dataset EV1, etc. Legends have to be provided in a separate tab in case of .xls files. Alternatively, the legend can be supplied as a separate text file (README) and zipped together with the Table/Dataset file.

11) The paper explained: EMBO Molecular Medicine articles are accompanied by a summary of the articles to emphasize the major findings in the paper and their medical implications for the non-specialist reader. Please provide a draft summary of your article highlighting

- the medical issue you are addressing,

- the results obtained and

- their clinical impact.

This may be edited to ensure that readers understand the significance and context of the research. Please refer to any of our published articles for an example.

12) Author contributions: You will be asked to provide CRediT (Contributor Role Taxonomy) terms in the submission system. These replace a narrative author contribution section in the manuscript.

13) A Conflict of Interest statement should be provided in the main text.

14) Every published paper includes a 'Synopsis' to further enhance discoverability. Synopses are displayed on the journal webpage and are freely accessible to all readers. They include a short stand first (maximum of 300 characters, including space) as well as 2-5 one-sentences bullet points that summarizes the paper. Please write the bullet points to summarize the key NEW findings. They should be designed to be complementary to the abstract - i.e. not repeat the same text. We encourage inclusion of key acronyms and quantitative information (maximum of 30 words / bullet point). Please use the passive voice. Please attach these in a separate file or send them by email, we will incorporate them accordingly.

Please also suggest a striking image or visual abstract to illustrate your article as a PNG file 550 px wide x 300 px high.

15) Include a Reagents and Tools Table as part of the Methods section, which can be downloaded from our author guidelines.

EMBO Molecular Medicine has a "scooping protection" policy, whereby similar findings that are published by others during review or revision are not a criterion for rejection. Should you decide to submit a revised version, I do ask that you get in touch after three months if you have not completed it, to update us on the status.

*Additional important information regarding Figures

Each figure should be given in a separate file and should have the following resolution:
Graphs 800-1,200 DPI
Photos 400-800 DPI
Colour (only CMYK) 300-400 DPI"

Figures are not edited by the production team. All lettering should be the same size and style; figure panels should be indicated by capital letters (A, B, C etc). Gridlines are not allowed except for log plots. Figures should be numbered in the order of their appearance in the text with Arabic numerals. Each Figure must have a separate legend and a caption is needed for each panel.

*Additional important information regarding figures and illustrations can be found at
https://media.springernature.com/original/springer-cms/rest/v1/content/27825798/data/v1

***** Reviewer's comments *****

Referee #1 (Comments on Novelty/Model System for Author):

Here the authors have entirely adressed my comments and strengthened their results.

Referee #1 (Remarks for Author):

The authors answered to the remaining issues which solidified their study. I have no more comment to add.

Referee #3 (Remarks for Author):

The authors have addressed adequately reviewers' comments

**EMM-2025-22759-V3**

**Editorial amendments**

Thank you for the submission of your revised manuscript to EMBO Molecular Medicine. I am pleased to inform you that we will be able to accept your manuscript pending the following final amendments:

1) Author Checklist: Please add corr. author name, journal and manusciprt number and make sure that all relevant questions are completed.

**We updated the file.**

2) In the main manuscript file, please do the following:
- Please address all comments suggested by our data editors listed below:
o Figure legends:
1. Please note that information related to n is missing in the legends of figures 2F, 3I.
2. Please note that the error bars are not defined in the legend of figure 2F.
3. Please define the annotated p values ****/***/**/* as well as provide the exact p-values for the same in the legend of figure 2F as appropriate.
4. Please note that the exact p values are not provided in the legends of figures 1A-C; 3E, F, G, H, J; 4E, F; 5A-C, D, E, F, G, J; 6C, EV1C, D, E; EV2 H, EV3 C, D, G, H, I, K, M; EV5 D, E, F, G, J, K.
5. Please indicate the statistical test used for data analysis in the legends of figures 2E, F; EV2 B, C.
6. Please note that the dashed borders are not defined in the legend of figure EV5 D. This needs to be rectified.

**We added all the missing information about "n" numbers and statistical tests used. The exact p-values are now in Table EV1.**

- The manuscript sections should be in the following order: Title page - Abstract - Introduction - Results - Discussion - Methods - Data Availability - Acknowledgments - Disclosure and Competing Interests Statement - References - Figure Legends - Main Tables with legends - Expanded View Figure Legends.

**The manuscript sections were reordered.**

- There is a callout for Fig. S2, please correct.

**We corrected the callout.**

- Please rename "Materials and Methods" to "Methods".

**We renamed the section.**

- In Methods, provide the antibody dilutions that were used for each antibody.

**We provided the antibody dilutions.**

- In Methods, provide the statement that experiments involving human subjects

conformed to the principles set out in the to the WMA Declaration of Helsinki and the Department of Health and Human Services Belmont Report.

**We added the statement.**

- In Methods, add the following paragraph:

Graphics:
(some of the... OR Figure #... OR synopsis) Graphics were created with BioRender.com.

**We added the Graphis paragraph in Methods section.**

- Indicate in legends exact n and exact p values, not a range, along with the statistical test used. To keep the figures "clear" some authors found providing an Appendix table Sx with all exact p-values preferable. You are welcome to do this if you want to.

**We added all the missing information about "n" numbers and statistical tests used. The exact p-values are now in Table EV1.**

- Author contributions: Please remove it from the manuscript and specify author contributions in our submission system. CRediT has replaced the traditional author contributions section because it offers a systematic machine-readable author contributions format that allows for more effective research assessment. You are encouraged to use the free text boxes beneath each contributing author's name to add specific details on the author's contribution. More information is available in our guide to authors:
https://www.embopress.org/page/journal/17574684/authorguide#authorshipguidelines

**We removed author contributions from the manuscript.**

- In data availability statement please use the following format to report the accession number of your data:

The datasets produced in this study are available in the following databases:
[data type]: [full name of the resource] [accession number/identifier] ([doi or URL or identifiers.org/DATABASE:ACCESSION])

**We added the links to database containing both proteomics datasets used in the study.**

Please check "Author Guidelines" for more information. https://www.embopress.org/page/journal/17574684/authorguide#availabilityofpublishedmaterial
- Please rename "Bibliography" to "References" and correct the reference citation in the text and reference list. In the text a reference should be cited by author and year of publication. Include a space between a word and the opening parenthesis of the reference that follows. In the reference list, citations should be listed in alphabetical order. Where there are more than 10 authors on a paper, 10 will be listed, followed by "et al.". DOIs should only be used for preprints and datasets that have not been published. Please check "Author Guidelines" for more information.

**We change the citation style in the manuscript.**

https://www.embopress.org/page/journal/17574684/authorguide#referencesformat
3) Funding: Please make sure that information about all sources of funding are complete in both our submission system and in the manuscript. Currently, core funding from Wellcome supporting Malawi-Liverpool-Wellcome Research Programme is missing in our submission system. Please correct.

**We added the missing funding information.**

4) The Paper Explained: Please add it to the main manuscript file and rename the subheadings to "Problem", "Results", "Impact".

**We added The Paper Explained section to the manuscript.**

5) Synopsis:
- Synopsis image: Please resize the image to exactly 550 px-wide x 300 pixels high and upload it as a high-resolution jpeg file.
- Synopsis text: Please remove "The Paper Explained" from the file.
- Please check your synopsis text and image before submission with your revised manuscript. Please be aware that in the proof stage minor corrections only are allowed (e.g., typos).

**We resized synopsis image to the correct resolution.**

6) As part of the EMBO Publications transparent editorial process (see our Editorial at http://embomolmed.embopress.org/content/2/9/329), EMBO Molecular Medicine will publish online a Review Process File (RPF) to accompany accepted manuscripts. This file will be published in conjunction with your paper and will include the anonymous referee reports, your point-by-point response and all pertinent correspondence relating to the manuscript. Let us know if you want to remove or not any figures from it prior to publication. Please note that the Authors checklist will be published at the end of the RPF.

**We do not wish to remove any figures from the point-by-point responses.**

7) Please provide a point-by-point letter INCLUDING my comments as well as the reviewer's reports and your detailed responses (as Word file).

11th May 2026

Dear Dr. Amulic,

We are pleased to inform you that your manuscript is accepted for publication and is now being sent to our publisher to be included in the next available issue of EMBO Molecular Medicine.

Your manuscript will be processed for publication by EMBO Press. It will be copy edited and you will receive page proofs prior to publication. Please note that you will be contacted by Springer Nature Author Services to complete licensing and payment information.

You may qualify for financial assistance for your publication charges - either via a Springer Nature fully open access agreement or an EMBO initiative. Check your eligibility: https://link.springer.com/journal/44321/how-to-publish-with-us

Should you be planning a Press Release on your article, please get in contact with embo_production@springernature.com as early as possible in order to coordinate publication and release dates.

If you have any questions, please do not hesitate to contact the Editorial Office. Thank you for your contribution to EMBO Molecular Medicine.

Yours sincerely,
Zeljko Durdevic

Zeljko Durdevic
Senior Editor
EMBO Molecular Medicine

------------------------------------------------

>>> Please note that it is EMBO Molecular Medicine policy for the transcript of the editorial process (containing referee reports and your response letter) to be published as an online supplement to each paper. If you do NOT want this, you will need to inform the Editorial Office via email immediately. More information is available here: https://link.springer.com/partners/embo-press/editorial-policies#Peer%20review

## EMBO Press Author Checklist

| | |
|---|---|
| Corresponding Author Name: Borko Amulic | |
| Journal Submitted to: EMBO Molecular Medicine | |
| Manuscript Number: EMM-2025-22759 | |

**USEFUL LINKS FOR COMPLETING THIS FORM**
The EMBO Journal - Author Guidelines
EMBO Reports - Author Guidelines
Molecular Systems Biology - Author Guidelines
EMBO Molecular Medicine - Author Guidelines

**Reporting Checklist for Life Science Articles (updated January**
This checklist is adapted from Materials Design Analysis Reporting (MDAR) Checklist for Authors. MDAR establishes a minimum set of requirements in transparent reporting in the life sciences (see Statement of Task: 10.31222/osf.io/9sm4x). Please follow the journal's guidelines in preparing your
**Please note that a copy of this checklist will be published alongside your article.**

**Abridged guidelines for figures**
**1. Data**
The data shown in figures should satisfy the following conditions:
- the data were obtained and processed according to the field's best practice and are presented to reflect the results of the experiments in an accurate and unbiased manner.
- ideally, figure panels should include only measurements that are directly comparable to each other and obtained with the same assay.
- plots include clearly labeled error bars for independent experiments and sample sizes. Unless justified, error bars should not be shown for technical
- if n<5, the individual data points from each experiment should be plotted. Any statistical test employed should be justified.
- Source Data should be included to report the data underlying figures according to the guidelines set out in the authorship guidelines on Data

**2. Captions**
Each figure caption should contain the following information, for each panel where they are relevant:
- a specification of the experimental system investigated (eg cell line, species name).
- the assay(s) and method(s) used to carry out the reported observations and measurements.
- an explicit mention of the biological and chemical entity(ies) that are being measured.
- an explicit mention of the biological and chemical entity(ies) that are altered/varied/perturbed in a controlled manner.
- the exact sample size (n) for each experimental group/condition, given as a number, not a range;
- a description of the sample collection allowing the reader to understand whether the samples represent technical or biological replicates (including how many animals, litters, cultures, etc.).
- a statement of how many times the experiment shown was independently replicated in the laboratory.
- definitions of statistical methods and measures:
  - common tests, such as t-test (please specify whether paired vs. unpaired), simple χ2 tests, Wilcoxon and Mann-Whitney tests, can be unambiguously identified by name only, but more complex techniques should be described in the methods section;
  - are tests one-sided or two-sided?
  - are there adjustments for multiple comparisons?
  - exact statistical test results, e.g., P values = x but not P values < x;
  - definition of 'center values' as median or average;
  - definition of error bars as s.d. or s.e.m.

**Please complete ALL of the questions below.**
**Select "Not Applicable" only when the requested information is not relevant for your study.**

**Materials**

| Newly Created Materials | Information included in the manuscript? | In which section is the information available? (Reagents and Tools Table, Materials and Methods, Figures, Data Availability Section) |
|---|---|---|
| New materials and reagents need to be available; do any restrictions apply? | Not Applicable | |

| Antibodies | Information included in the manuscript? | In which section is the information available? (Reagents and Tools Table, Materials and Methods, Figures, Data Availability Section) |
|---|---|---|
| For **antibodies** provide the following information:<br>- Commercial antibodies: RRID (if possible) or supplier name, catalogue number and or/clone number<br>- Non-commercial: RRID or citation | Yes | Reagents and Tools Table |

| DNA and RNA sequences | Information included in the manuscript? | In which section is the information available? (Reagents and Tools Table, Materials and Methods, Figures, Data Availability Section) |
|---|---|---|
| **Short novel DNA or RNA including primers, probes**: provide the sequences. | Not Applicable | |

| Cell materials | Information included in the manuscript? | In which section is the information available? (Reagents and Tools Table, Materials and Methods, Figures, Data Availability Section) |
|---|---|---|
| **Cell lines:** Provide species information, strain. Provide accession number in repository **OR** supplier name, catalog number, clone number, and/**OR** RRID. | Yes | Reagents and Tools Table |
| **Primary cultures:** Provide species, strain, sex of origin, genetic modification status. | Not Applicable | |
| Report if the cell lines were recently **authenticated** (e.g., by STR profiling) and tested for mycoplasma contamination. | Not Applicable | |

| Experimental animals | Information included in the manuscript? | In which section is the information available? (Reagents and Tools Table, Materials and Methods, Figures, Data Availability Section) |
|---|---|---|
| **Laboratory animals or Model organisms:** Provide species, strain, sex, age, genetic modification status. Provide accession number in repository **OR** supplier name, catalog number, clone number, **OR** RRID. | Yes | Methods |
| **Animal observed in or captured from the field:** Provide species, sex, and age where possible. | Not Applicable | |
| Please detail **housing and husbandry conditions**. | Yes | Methods |

| Plants and microbes | Information included in the manuscript? | In which section is the information available? (Reagents and Tools Table, Materials and Methods, Figures, Data Availability Section) |
|---|---|---|
| **Plants:** provide species and strain, ecotype and cultivar where relevant, unique accession number if available, and source (including location for collected wild specimens). | Not Applicable | |
| **Microbes:** provide species and strain, unique accession number if available, and source. | Not Applicable | |

| Human research participants | Information included in the manuscript? | In which section is the information available? (Reagents and Tools Table, Materials and Methods, Figures, Data Availability Section) |
|---|---|---|
| If collected and within the bounds of privacy constraints report on age, sex and gender or ethnicity for all study participants. | Yes | Methods; Table 1 and 2 |

| Core facilities | Information included in the manuscript? | In which section is the information available? (Reagents and Tools Table, Materials and Methods, Figures, Data Availability Section) |
|---|---|---|
| If your work benefited from core facilities, was their service mentioned in the acknowledgments section? | Yes | Acknowledgments |

**Design**

| Study protocol | Information included in the manuscript? | In which section is the information available? (Reagents and Tools Table, Materials and Methods, Figures, Data Availability Section) |
|---|---|---|
| If study protocol has been **pre-registered, provide DOI in the manuscript**. For clinical trials, provide the trial registration number **OR** cite DOI. | Not Applicable | |
| Report the **clinical trial registration number** (at ClinicalTrials.gov or equivalent), where applicable. | Not Applicable | |

| Laboratory protocol | Information included in the manuscript? | In which section is the information available? (Reagents and Tools Table, Materials and Methods, Figures, Data Availability Section) |
|---|---|---|
| Provide DOI OR other citation details if **external detailed step-by-step protocols** are available. | Not Applicable | |

| Experimental study design and statistics | Information included in the manuscript? | In which section is the information available? (Reagents and Tools Table, Materials and Methods, Figures, Data Availability Section) |
|---|---|---|
| Include a statement about **sample size** estimate even if no statistical methods were used. | Yes | Figure Legends |
| Were any steps taken to minimize the effects of subjective bias when allocating animals/samples to treatment (e.g. **randomization procedure**)? If yes, have they been described? | Yes | Methods |
| Include a statement about **blinding** even if no blinding was done. | Yes | Methods |
| Describe **inclusion/exclusion criteria** if samples or animals were excluded from the analysis. Were the criteria pre-established?<br><br>If sample or data points were omitted from analysis, report if this was due to attrition or intentional exclusion and provide justification. | Yes | Methods |
| For every figure, are **statistical tests** justified as appropriate? Do the data meet the assumptions of the tests (e.g., normal distribution)? Describe any methods used to assess it. Is there an estimate of variation within each group of data? Is the variance similar between the groups that are being statistically compared? | Yes | Figure Legends |

| Sample definition and in-laboratory replication | Information included in the manuscript? | In which section is the information available? (Reagents and Tools Table, Materials and Methods, Figures, Data Availability Section) |
|---|---|---|
| In the figure legends: state number of times the experiment was **replicated** in laboratory. | Yes | Figure Legends |
| In the figure legends: define whether data describe **technical or biological replicates**. | Yes | Materials and Methods, Figure Legends |

## Ethics

| Ethics | Information included in the manuscript? | In which section is the information available? (Reagents and Tools Table, Materials and Methods, Figures, Data Availability Section) |
|---|---|---|
| Studies involving **human participants**: State details of **authority granting ethics approval** (IRB or equivalent committee(s), provide reference number for approval. | Yes | Methods |
| Studies involving **human participants**: Include a statement confirming that **informed consent** was obtained from all subjects and that the experiments conformed to the principles set out in the WMA Declaration of Helsinki and the Department of Health and Human Services Belmont Report. | Yes | Methods |
| Studies involving **human participants:** For publication of **patient photos**, include a statement confirming that consent to publish was obtained. | Not Applicable | |
| Studies involving experimental **animals**: State details of **authority granting ethics approval** (IRB or equivalent committee(s), provide reference number for approval. Include a statement of compliance with ethical regulations. | Yes | Methods |
| Studies involving **specimen and field samples:** State if relevant **permits** obtained, provide details of authority approving study; if none were required, explain why. | Not Applicable | |

| Dual Use Research of Concern (DURC) | Information included in the manuscript? | In which section is the information available? (Reagents and Tools Table, Materials and Methods, Figures, Data Availability Section) |
|---|---|---|
| Could your study fall under dual use research restrictions? Please check biosecurity documents and list of **select agents and toxins** (CDC): https://www.selectagents.gov/sat/list.htm | Not Applicable | |
| If you used a select agent, is the security level of the lab appropriate and reported in the manuscript? | Not Applicable | |
| If a study is subject to dual use research of concern regulations, is the name of the **authority granting approval and reference number** for the regulatory approval provided in the manuscript? | Not Applicable | |

## Reporting

The MDAR framework recommends adoption of discipline-specific guidelines, established and endorsed through community initiatives. Journals have their own policy about requiring specific guidelines and recommendations to complement MDAR.

| Adherence to community standards | Information included in the manuscript? | In which section is the information available? (Reagents and Tools Table, Materials and Methods, Figures, Data Availability Section) |
|---|---|---|
| State if relevant guidelines or checklists (e.g., **ICMJE, MIBBI, ARRIVE, PRISMA**) have been followed or provided. | Not Applicable | |
| For **tumor marker prognostic studies,** we recommend that you follow the **REMARK** reporting guidelines (see link list at top right). See author guidelines, under 'Reporting Guidelines'. Please confirm you have followed these guidelines. | Not Applicable | |
| For **phase II and III randomized controlled trials**, please refer to the **CONSORT** flow diagram (see link list at top right) and submit the CONSORT checklist (see link list at top right) with your submission. See author guidelines, under 'Reporting Guidelines'. Please confirm you have submitted this list. | Not Applicable | |

## Data Availability

| Data availability | Information included in the manuscript? | In which section is the information available? (Reagents and Tools Table, Materials and Methods, Figures, Data Availability Section) |
|---|---|---|
| Have **primary datasets** been deposited according to the journal's guidelines (see 'Data Deposition' section) and the respective accession numbers provided in the Data Availability Section? | Yes | Data Availability |
| Were **human clinical and genomic datasets** deposited in a public access-controlled repository in accordance to ethical obligations to the patients and to the applicable consent agreement? | Not Applicable | |
| Are **computational models** that are central and integral to a study available without restrictions in a machine-readable form? Were the relevant accession numbers or links provided? | Not Applicable | |
| If publicly available data were reused, provide the respective **data citations in the reference list**. | Not Applicable | |
